# Impaired Glymphatic Function and Pulsation Alterations in a Mouse Model of Vascular Cognitive Impairment

**DOI:** 10.3389/fnagi.2021.788519

**Published:** 2022-01-13

**Authors:** Mosi Li, Akihiro Kitamura, Joshua Beverley, Juraj Koudelka, Jessica Duncombe, Ross Lennen, Maurits A. Jansen, Ian Marshall, Bettina Platt, Ulrich K. Wiegand, Roxana O. Carare, Rajesh N. Kalaria, Jeffrey J. Iliff, Karen Horsburgh

**Affiliations:** ^1^Centre for Discovery Brain Sciences, University of Edinburgh, Edinburgh, United Kingdom; ^2^Edinburgh Medical School, UK Dementia Research Institute, University of Edinburgh, Edinburgh, United Kingdom; ^3^Department of Neurology, Shiga University of Medical Science, Otsu, Japan; ^4^Centre for Cardiovascular Science, University of Edinburgh, Edinburgh, United Kingdom; ^5^Centre for Clinical Brain Sciences, University of Edinburgh, Edinburgh, United Kingdom; ^6^School of Medicine, Medical Sciences and Nutrition, College of Life Sciences and Medicine, University of Aberdeen, Aberdeen, United Kingdom; ^7^Faculty of Medicine, University of Southampton, Southampton, United Kingdom; ^8^Translational and Clinical Research Institute, Newcastle University, Newcastle upon Tyne, United Kingdom; ^9^VISN 20 Mental Illness Research, Education and Clinical Center, VA Puget Sound Health Care System, Seattle, WA, United States; ^10^Department of Psychiatry and Behavioral Sciences, University of Washington School of Medicine, Seattle, WA, United States; ^11^Department of Neurology, University of Washington School of Medicine, Seattle, WA, United States

**Keywords:** carotid stenosis, vascular pulsation, glymphatic function, vascular cognitive impairment, amyloid-β (Aβ), cerebral amyloid angiopathy (CAA)

## Abstract

Large vessel disease and carotid stenosis are key mechanisms contributing to vascular cognitive impairment (VCI) and dementia. Our previous work, and that of others, using rodent models, demonstrated that bilateral common carotid stenosis (BCAS) leads to cognitive impairment via gradual deterioration of the neuro-glial-vascular unit and accumulation of amyloid-β (Aβ) protein. Since brain-wide drainage pathways (glymphatic) for waste clearance, including Aβ removal, have been implicated in the pathophysiology of VCI via glial mechanisms, we hypothesized that glymphatic function would be impaired in a BCAS model and exacerbated in the presence of Aβ. Male wild-type and Tg-SwDI (model of microvascular amyloid) mice were subjected to BCAS or sham surgery which led to a reduction in cerebral perfusion and impaired spatial learning acquisition and cognitive flexibility. After 3 months survival, glymphatic function was evaluated by cerebrospinal fluid (CSF) fluorescent tracer influx. We demonstrated that BCAS caused a marked regional reduction of CSF tracer influx in the dorsolateral cortex and CA1-DG molecular layer. In parallel to these changes increased reactive astrogliosis was observed post-BCAS. To further investigate the mechanisms that may lead to these changes, we measured the pulsation of cortical vessels. BCAS impaired vascular pulsation in pial arteries in WT and Tg-SwDI mice. Our findings show that BCAS influences VCI and that this is paralleled by impaired glymphatic drainage and reduced vascular pulsation. We propose that these additional targets need to be considered when treating VCI.

## Introduction

Cerebral vascular disease (CVD) is a major contributor to vascular cognitive impairment (VCI) and dementia such as Alzheimer’s disease (Gorelick et al., 2011; Montine et al., 2014). Large well-characterized cohort studies have highlighted the co-existence of vascular disease with Alzheimer’s disease (De Jong et al., 1997; Hachinski and Munoz, 1997; Snowdon et al., 1997; Esiri et al., 1999; de la Torre, 2000a,b,c). Key neuroimaging features (white matter lesions, microbleeds, lacunes and perivascular spaces) are found in both Alzheimer’s disease and VCI sharing a number of vascular risk factors, such as hypertension, diabetes and atherosclerosis (Dichgans and Leys, 2017). Vascular risk factors in midlife are also associated with increased burden of Alzheimer-related pathology, such as amyloid protein, suggesting a direct relationship (Gottesman et al., 2017).

Chronic cerebral hypoperfusion has been proposed as a central common mechanism which contributes to cognitive decline and degenerative processes leading to dementia (Duncombe et al., 2017a). Global reductions in blood flow are associated with increased risk of progression from mild cognitive impairment to dementia suggesting that perfusion plays a key role in disease progression (Alsop et al., 2010; Chao et al., 2010). Reduced cerebral perfusion has been linked to white matter attenuation, a key feature common to both Alzheimer’s disease and dementia associated with CVD (Schuff et al., 2009; Barker et al., 2014). Common artery stenosis of varying degrees is invariably associated with cognitive impairment (Johnston et al., 2004; Cheng et al., 2012; Alosco et al., 2013; Balestrini et al., 2013) and carotid stenosis (> 25%) has been linked to a greater burden of white matter hyperintensities (Romero et al., 2009). Large and small vessel disease is also linked to Alzheimer’s disease dementia (Arvanitakis et al., 2016). Reduced cerebral perfusion, impaired cerebrovascular reactivity and hemodynamic responses are increasingly recognized in the early stages of Alzheimer’s disease (de la Torre, 2012b; Hughes et al., 2014). Our work and others using animal models have shown that chronic cerebral hypoperfusion as a result of bilateral carotid stenosis leads to cognitive decline through mechanisms that involve hypoxia-induced white matter damage and gradual deterioration of the neuro-glial-vascular unit including endothelial dysfunction, microvascular inflammation and BBB leakage (Shibata et al., 2004; Holland et al., 2015; Fowler et al., 2017; Kitamura et al., 2017; Roberts et al., 2018). There is substantial evidence that reduced blood flow contributes to vascular disease. However, a causal relationship remains a matter of controversy largely due to the cross-sectional nature of clinical studies and, in the few longitudinal studies conducted, reduced blood flow occurs subsequent to vascular disease burden (de la Torre, 2012a). Impaired glymphatic function is emerging as a key player in vascular disease and dementia. The glymphatic pathway is a brain wide clearance process that relies on the movement of CSF along the perivascular network facilitated by aquaporin-4 water channels on the astroglial endfeet to promote the elimination of waste out of the brain (Iliff et al., 2012). CSF flow within the perivascular space (PVS) is regulated by cerebrovascular pulsatility and constriction, which is now considered to be a key factor regulating glymphatic function (Iliff et al., 2013b; Mestre et al., 2018, 2020). Enlarged PVS, identified by neuroimaging, are a common feature of CVD and dementia linked to vascular risk factors and inflammation (Doubal et al., 2010; Wardlaw et al., 2013; Aribisala et al., 2014b; Potter et al., 2015; Shi and Wardlaw, 2016; Ding et al., 2017). There is also evidence of impaired glymphatic function in pre-clinical models relevant to CVD. Notably advanced age, acute ischemic stroke and multi- infarct stroke, diabetes and subarachnoid hemorrhage (SAH) have all been shown to have a major impact on glymphatic drainage (Gaberel et al., 2014; Kress et al., 2014; Wang et al., 2017). Disturbances of the glymphatic function are also related to a build-up of Aβ in both human and rodent brain (Xu et al., 2015; Shokri-Kojori et al., 2018).

Recent studies from our group and others have shown that carotid stenosis reduces cerebral perfusion and alters the Aβ peptide pools culminating in cerebral amyloid angiopathy (CAA) and vascular related lesions (Okamoto et al., 2012; Salvadores et al., 2017). In light of the evidence that flow-limiting large-vessel stenosis contributes to vascular and Alzheimer’s disease pathophysiology (Gupta and Iadecola, 2015), and that impaired glymphatic function is a key contributor to impaired Aβ clearance we hypothesized that the complex interaction of Alzheimer’s disease and carotid stenosis leading to cognitive impairment occurs via impaired glymphatic function in addition to perfusion deficits. We interrogated this by examining glymphatic influx in a well-characterized murine model of VCI induced by bilateral common carotid stenosis (BCAS) (Shibata et al., 2004) and then assessed Aβ accumulation in a model of microvascular amyloid (Tg-SwDI) post-BCAS. We further assessed astrocytes and cerebral vascular pulsation as potential mechanisms since they govern CSF-ISF exchange in murine brain (Iliff et al., 2013b).

## Materials and Methods

### Mice

All experiments were conducted in accordance with the United Kingdom Home Office Animals (Scientific Procedures) Act 1986 and additional local ethical and veterinary approval (Biomedical Research Resources, University of Edinburgh) and the ARRIVE guidelines. We used male C57Bl/6J (Charles River Laboratories Inc., United Kingdom) and Tg-SwDI mice (transgenic mice with Swedish, Dutch and Iowa mutations in human amyloid precursor protein (APP), with primarily microvascular amyloidosis) for all experiments. At the outset, mice from cohort 1 (*n* = 42) (Tg-SwDI and wild-type littermates at 7–9 months old) were randomly assigned to experiments of MRI and behavioral tests, and tissues were collected for evaluation of microvascular amyloid level. A second cohort of mice (cohort 2) (*n* = 33) (Tg-SwDI at 5–7 months and imported C57Bl/6J mice at 4–5 months) were used for the investigation of glymphatic influx and astrogliosis. Mice from cohort 3 were used for *in vivo* investigation of vessel pulsation. Investigators were blinded to surgery and genotype throughout the data collection and analysis. Final group size for analysis: cohort 1: *n* = 8 WT sham, *n* = 10 WT BCAS, *n* = 6 Tg-SwDI sham, *n* = 10 Tg-SwDI BCAS. Cohort 2, *n* = 10 WT sham, *n* = 8 WT BCAS, *n* = 7 Tg-SwDI sham, *n* = 8 Tg-SwDI BCAS. Cohort 3, *n* = 7 WT sham, *n* = 7 WT BCAS, *n* = 6 Tg-SwDI sham, *n* = 7 Tg-SwDI BCAS.

### Bilateral Common Carotid Stenosis Surgery

BCAS surgery was performed under isoflurane anesthesia by applying microcoils (0.18 mm internal diameter, Sawane Spring Co, Shizuoka, Japan) permanently to both common carotid arteries. Details of surgical methods have been described in previous studies (Shibata et al., 2004; Coltman et al., 2011; Holland et al., 2011; Reimer et al., 2011). A 30-min interval was given between two microcoils application to minimize the acute CBF changes caused by the placement of microcoils. Sham-operated animals underwent the identical procedure except the application of microcoils to both arteries. In cohort 1, one Tg-SwDI mouse was culled during surgery due to severe bleeding; and after 3 days of surgery, two WT and five Tg-SwDI were culled due to poor recovery as their weight loss exceeded 20%. Therefore, these mice were excluded from the study.

### Cerebral Blood Flow Measure by Arterial Spin Labeling

A 7.0T (Agilent Technologies, Yarnton, United Kingdom) preclinical MRI system was used to collect T1-weighted and arterial spin labeling (ASL) data as we previously described (Duncombe et al., 2017b). Experimental animals were anesthetized under 5% isoflurane in oxygen for induction then placed in an MRI compatible holder (Rapid Biomedical, Wurzburg, Germany). Isoflurane was maintained at 1.5% in oxygen during scanning. Rectal temperature was monitored and regulated at around 37°C by an airflow heating system. Respiratory rate was regulated at 70–100 breaths per minute. The T1-weighted images were acquired at 1.7 mm posterior to Bregma in stereotactic coordinates of Mouse Brain Atlas (Paxinos and Franklin, 2001). ASL was performed using a Look-Locker FAIR single gradient echo (LLFAIRGE) sequence (Kober et al., 2008) covering a 1.5 mm thick brain slice centered –1.7 mm posterior from Bregma. Forty gradient echoes spaced 200 ms apart were acquired after a slice-selective or global adiabatic inversion pulse for each phase encoding, resulting in a total observation time of approximately 16 min for a 64 × 64 imaging matrix. The flip angle was 20°. The first 20° pulse occurred 3 ms after the inversion pulse. The echo time was 1.42 ms. Maps of cerebral blood flow (CBF) were constructed from ASL data in MATLAB using in-house scripts. CBF maps were analyzed in ImageJ (v1.46, NIH, Bethesda, MD, United States) using unbiased and uniform regions of interest from T1-weighted images acquired with the ASL sequence. The CBF values in each region of interest were reported as % change compared to baseline.

### Assessment of Cognitive Function Using Barnes Maze

**FIGURE 1:**
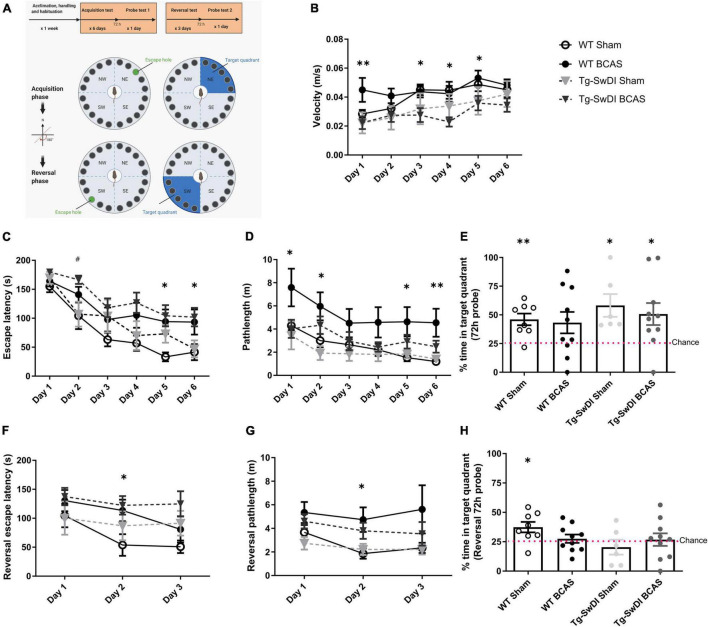
BCAS causes a decline in spatial learning acquisition and cognitive flexibility. Spatial learning and memory and cognitive flexibility were assessed using a Barnes maze at 3 months post-BCAS in WT and Tg-SwDI mice. **(A)** In the acquisition phase one hole (indicated by green) was designated as the target hole with an escape box. A probe test was performed 72 h after the last acquisition training session, in which the escape box was removed. In the reversal phase, the target hole was moved 180 degrees to the original target hole. 1 day after the probe test. A reversal probe test was performed 72 h after the last training session. **(B)** Motor ability assessment in acquisition training. The speed or velocity was measured at the outset to assess whether motor function was affected by the genotype or surgery across different groups. There was a significant effect of genotype between WT and Tg-SwDI mice. *Post hoc* tests showed significant difference between WT BCAS and Tg-SwDI BCAS mice at day 1 (^**^p < 0.01), 3, 4 and 5 (*p < 0.05), respectively. **(C)** Spatial learning was assessed by comparing escape latency over 6 days with 2 sessions per day. There was a significant effect of BCAS surgery but not genotype across groups. *Post hoc* tests showed significant effect of BCAS in WT at day 5, 6 (*p < 0.05) and in Tg-SwDI at day 2 (^#^*p* < 0.05) compared to their sham counterparts. **(D)** Pathlength measure was also used to evaluate spatial learning function. There was a significant effect of BCAS surgery but not genotype across groups. *Post hoc* tests showed significant effect of BCAS in WT mice when compared to their sham counterparts at day 1, 2, 5 (*p < 0.05) and 6 (^**^p < 0.01). **(E)** In the acquisition 72 h probe test all mice performed above chance except WT BCAS mice (one sample *t*-test). No significant effect of BCAS and genotype were detected (Two-way ANOVA). To enhance the detection of spatial learning ability, reversal trials were taken to evaluate the ability of mice to learn a new location using Barnes maze **(F–H)**. In the reversal tests, spatial learning was assessed by comparing escape latency and pathlength over 3 days with 2 sessions per day training across all groups. **(F)** There was a significant effect of BCAS surgery but not genotype by comparing escape latency. *Post hoc* tests showed significant effect of BCAS in WT mice when compared to their sham counterparts at day 2 (*p < 0.05). **(G)** By comparing the pathlength in the test, there was a significant effect of BCAS surgery but not genotype. *Post hoc* tests showed significant effect of BCAS in WT mice when compared to their sham counterparts at day 2 (*p* < 0.05). **(H)** In the reversal probe test only WT sham mice performed above chance (one sample *t*-test). There was no significant effect of either genotype or surgery on the percentage time spent in the correct quadrant (Two-way ANOVA). Data are mean ± SEM, *n* = 6–10 per group.

A Barnes maze was used to assess the differences in spatial learning and memory at 3 months after BCAS or sham surgery (Barnes maze schedule shown in Figure 1A). The maze consists of one white circular platform and 20 circular holes around the outside edge of the platform, with 91.5 cm diameter and 115 cm height (San Diego Instruments). The maze was brightly lit with lamps and overhead room lights (450 lux), and an aversive white noise stimulus is played at 85 dB. There is one dark escape chamber attached to one of the holes allocated to each experimental animal. Visual cues were placed on the curtains and walls around the maze. There was one white cylinder with 10.5 cm diameter for retaining animals at the beginning of each trial. All the tests were recorded by a video-based automatic tracking system ANY-maze v 4.99. All the tests were performed in the behavior testing room where the room temperature can be controlled at constant 20°C.

#### Acclimation and Habituation

Animals were brought into the behavioral testing room and placed in the holding cylinder to acclimate to the testing environment for 10 s for 2 days before habituation. One week prior to the training session, animals were habituated to the maze and escape chamber. Each mouse was placed in the holding cylinder for 10 s then allowed 3 min free exploration under low stress conditions after removal of the cylinder, without aversive white noise stimulation. Then mice were guided to the escape chamber and allowed inside for 2 min. All the animals were allocated one fixed number for the chamber during the behavior test. The maze and the escape chamber were cleaned with ethanol to avoid any olfactory cues between each trial.

#### Visuo-Spatial Learning and Working Memory Test (Acquisition Training)

During the training session, mice were trained to find the escape chamber over 6 days with 2 trials per day (60-min inter-trial interval). The platform consists of 20 escape holes and the location of escape chamber remained constant to each mouse but was shifted clockwise 90 degree between mice to avoid any olfactory cues. The mouse was placed in the holding cylinder for 10 s. The aversive white noise (85 dB) was given once the test started and switched off once the mouse entered the escape chamber. If the mouse failed to enter the target hole, the experimenter guided the mouse to the escape chamber. The aversive stimulus was stopped as soon as the mouse entered the chamber.

#### 72 h Probe

A probe trial was performed 72 h after the final acquisition training and each mouse was allowed 90 s to explore the maze with the escape chamber removed and the rest of elements remained same. The 72 h probe trials aimed to test the long-term memory of the mice after a period of training to locate the escape chamber.

#### Reversal Training

During the reversal training session, mice were trained to find the escape chamber following same procedure as the acquisition training phase, but with the allocated escape chamber shifted 180 degree to the opposite side of the stage. The mice were trained over 3 days with 2 trials per day (60-min inter-trial interval) in reversal training to evaluate the spatial learning ability in increased difficulty of task.

#### Reversal Probe

The reversal probe trial was performed 72 h after the final reversal training. Animals were given 90 s to explore the maze with the escape chamber removed. All the elements in reversal probe remained same as reversal training test.

#### Measurements

Trials were recorded by a camera above the maze and measured using tracking software Any-maze version 4.99. Spatial learning was assessed by the total time to enter the escape chamber (escape latency) and the total distance traveled (pathlength) during this period.

### Assessment of Glymphatic Function by Intracisternal Injection of Fluorescent Tracers

Mice were initially anesthetized with isoflurane (5% in oxygen), then positioned on a stereotaxic frame and anesthetic maintained at approximately 1.5% (in oxygen). The respiration was regulated using a ventilator. The posterior atlanto-occipital membrane was surgically exposed and a 32GA needle attached to a Hamilton syringe was inserted into cisterna magna. Dextran, fluorescein and biotin labeled 3 kDa soluble lysine fixable (D7156, Invitrogen) and ovalbumin Alexa Fluor^®^ 594 conjugate 45 kDa (O34783, Invitrogen) tracers were mixed at 1:1 ratio and infused at a concentration of 5 μg/μl, at a rate of 0.5 μl/min over 20 min (10 μl total volume) through a syringe pump (Harvard Apparatus). The needle was held in place for 10 min and then removed, and atlanto-occipital membrane was sealed to avoid any reflux of CSF.

### Tissue Processing

At the end of the experiments, mice from cohort 1 and 2 were transcardially perfused with 30 ml heparinized saline then whole brains were fixed in 4% paraformaldehyde in PBS for 24 h. For cohort 1, brain tissues were further transferred into 30% sucrose solution in PBS for 72 h. Brains were placed in pre-cool isopentane –42°C for 5 min then stored in –80°C freezer and coronal sections (12 μm) were cut using a cryostat. For cohort 2, the brains were sectioned into coronal planes (100 μm) on a vibratome then stored in cryoprotective medium in a –20°C freezer.

### Imaging of Fluorescent Tracer Movement

Tracer movement from the subarachnoid space of the cisterna magna into the brain was imaged using a slide scanner (ZEISS Axio Scan.Z1). Multi-channel whole-slice images of each animal at hippocampal level (–1.82 mm to bregma, Mouse Brain Atlas) was generated at 20 × magnification. This included separate DAPI, Alexa Fluor 488 and Alexa Fluor 594 channels. All images were scanned using constant exposure time for each individual channel by the slide scanner. For the quantification of tracer movement into the brain, scanned images were analyzed in ImageJ software (v1.46, NIH, Bethesda, MD, United States) as described previously (Iliff et al., 2012). Region of interest (ROI) was defined using DAPI channel to identify anatomical regions. Auto-thresholding (triangle method) was used to measure the % area of positive signal that is the glymphatic CSF influx.

### Immunohistochemistry

Immunostaining was carried out according to standard protocols. Frozen sections were removed from the freezer and allowed to air dry for 30 min. Slides were washed in phosphate-buffered saline (PBS) followed by a series of ethanol (70, 90, and 100%) for dehydration then placed in xylene for 10 min. Sections were rehydrated through serial ethanol (100, 90, and 70%) then rinsed in water. Antigen retrieval was performed using 10 mM citric buffer (PH 6.0) at 100°C under pressure for 10 min then covered with proteinase K working solution for 10 min at room temperature. Sections were rinsed in PBS and incubated in blocking buffer (10% normal serum, 0.5% BSA) for 1 h at room temperature. Subsequently, sections were incubated in primary antibody solution (amyloid 6E10, 1:1,000, Covance, SIG-39320, mouse monoclonal antibody; COL4, 1:400, Fitzgerald, 70R-CR013X, rabbit polyclonal antibody) overnight at 4°C. Sections were then rinsed in PBS and incubated in secondary antibody (anti-rabbit Alexa Fluor 546, 1:500, Invitrogen A-11010; anti-mouse Alexa Fluor 488 1:500, Invitrogen A-11001) for 1 h at room temperature.

Vibratome sections were rinsed in PBS and mounted on to superfrost plus slides (VWR international) followed by serial ethanol (70, 90, and 100%) and then placed in xylene for 10 min. Sections were rehydrated through serial ethanol (100, 90, and 70%) then rinsed in running water. Antigen retrieval was performed using 10 mM citric buffer (PH 6.0) at 100°C under pressure for 10 min. Then sections were incubated in primary antibody solution (GFAP, 1:1,000, Life technologies, 13-0300, Rat monoclonal antibody) overnight at 4°C. Sections were rinsed in PBS and incubated in non-fluorescent biotinylated secondary antibody (anti-rat, 1:100, Vector Laboratories, YO809) for 1 h at room temperature followed by 1 h incubation with Vector ABC Elite kit (Vector Laboratories). Finally, sections were visualized with DAB peroxidase substrate kit (Vector Laboratories).

### Analysis of Immunohistochemistry

Immunostained 12 μm frozen sections were analyzed using a laser scanning confocal microscope (ZEISS LSM 710, Germany). Cortical amyloid load and blood vessel density were determined by measuring the percentage of areas occupied by 6E10 and COL4 staining, respectively. Vascular amyloid load was determined by colocalization analysis for blood vessels and amyloid by calculating the Mander’s coefficient and data shown as % vascular amyloid. Images from the cortex in Tg-SwDI mice were selected, amyloid, blood vessels (COL4) and vascular amyloid images were quantified at the brain regions from the pial surface to approximate the depth of 250 μm. Immunostained 100 μm thick vibratome sections were analyzed using a slide scanner (ZEISS Axio Scan.Z1). Astrogliosis were assessed by measuring the percentage of stained area occupied by GFAP staining, using auto thresholding (triangle method). All measurements were carried out using ImageJ (v1.46, NIH, Bethesda, MD, United States).

### Cranial Window Implantation

Animals were initially anesthetized using 4–5% isoflurane, delivered through a face mask via a ventilator and kept on 1.5–2% through the surgery. Subcutaneous injection of Caprofen (5 mg/kg) was administered at the start of the surgery. Body temperature was monitored through the surgery. Skin was removed to expose the skull, dried, and secured using VetBond (3M, #1649). Using a high-speed micro drill, an area of 6 × 3 mm was drilled over until a thin layer of bone was left. A drop of artificial cerebrospinal fluid (ACSF) (ACSF; 125 mM NaCl, 10 mM glucose, 10 mM HEPES, 3.1 mM CaCl_2_, 1.3 mM MgCl_2_, pH 7.4), was applied to the skull and left for 10 min. Using angled forceps, the skull was lifted without disrupting the dura. Hemocollagene soaked in ACSF was applied on the exposed brain for 5 min. A sterile coverslip was placed on top of the exposed brain, and secured by a mixture of liquid glue and dental cement. Immediately afterward, a custom made head plate (Protolabs) was applied to the cranial window prep and secured by additional glue/cement mixture. The cranial window was left to dry for 5 min and the animal was placed for recovery. Animals were rested for 4 weeks prior to imaging.

### *In vivo* Vascular Pulsation Assessment

In separate cohorts of 7–8 month old WT and Tg-SwDI mice (cohort 3) cerebral vascular pulsatility was evaluated in WT and Tg-SwDI mice 1 month after sham and BCAS surgery. Cerebral vascular pulsation was assessed through the cortical vascular network with the use of multiphoton microscopy (LaVision Biotech TriMScope with Nikon CFI-Apo 25 × NA1.1 lens and Leica SP8 DIVE with IRAPO L 25 × NA1.0 lens) using a method based on those described previously within the literature (Iliff et al., 2013b; Kress et al., 2014). Mice were anesthetized in 5% isoflurane and maintained at 1.5–2% through imaging. Cortical vascular network was visualized via multiphoton microscopy through the injection of fluorescently conjugated dextran into the blood stream (Rhodamine B, 20 mg/ml, Sigma R9379). The pulsatility of individual vessels was determined by positioning linescans orthogonal to the vessel’s axis. Linescans were performed in a repeated loop scans at a frequency of 1086.96 Hz (LaVision Biotech TriMScope) and 8,000 Hz (Leica SP8 DIVE) to equal duration of 3,600 ms. To calculate dynamic vessel width changes over time, the kymograph resulting from line scans was smoothed in FIJI (Smooth 3D) and thresholded (default automatic threshold). Using ROI placement over the whole graph, the distribution of pixel density for each line was calculated using a multi-plot. After conversion of pixels to μms and ms, the vessel width (μm) within each region was plotted against time (ms). Vessel wall pulsatility (μm*ms) was calculated from the resulting graphs as the absolute value of area under the diameter-time plot, integrated to the running average calculated across the entire 3,600 ms sampling time (GraphPad Prism, AUC analysis).

### Statistical Analysis

Data were analyzed using a two-way ANOVA with surgery and genotype as two between-subject factors followed by Bonferroni’s multiple comparison test to compare CBF levels, CSF glymphatic drainage and astrogliosis. Statistical comparison of spatial learning was carried out by repeated measures ANOVA with surgery and genotype as between subject factors followed by Bonferroni’s multiple comparison test, the probe trials were carried out by using two-way ANOVA for comparison between groups. One sample *t*-test was used to compare the performance of each group with the chance. Mann Whitney *U*-test was used to compare the amyloid burden, blood vessel density and pulsation. Statistical analysis was performed using IBM SPSS Statistics 22.

## Results

### Regional Cerebral Perfusion Is Reduced Post- Bilateral Common Carotid Stenosis in Wild-Type and Tg-SwDI Mice

**FIGURE 2:**
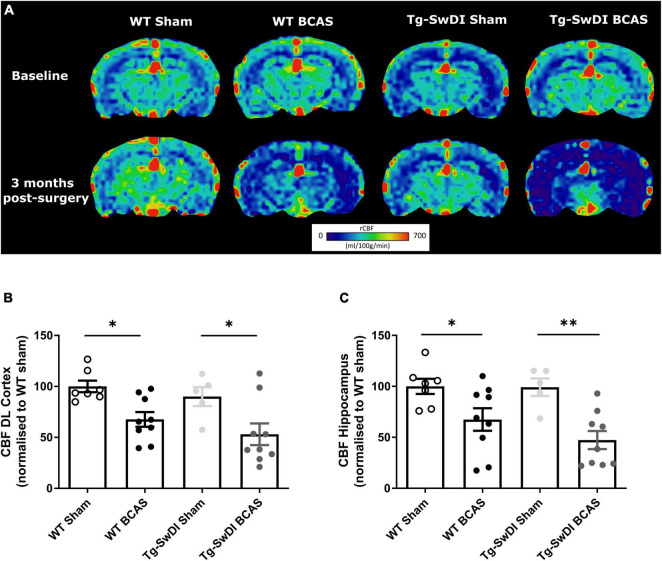
Decreased resting CBF following BCAS. MRI arterial spin labeling (ASL) was used to measure regional alterations in CBF. **(A)** Representative images of arterial spin labeling (ASL) from sham and BCAS WT and Tg-SwDI mice at 3 months following surgery. **(B,C)** A significant reduction of CBF in the brain cortex and hippocampus was determined post-BCAS but there was no genotype effect. * and ^**^ indicate *p* < 0.05 and 0.01, respectively. Data are presented as individual data points, mean ± SEM, *n* = 6–10 per group.

At the outset of the studies, we determined whether carotid stenosis affected cerebral blood flow (CBF) in Tg-SwDI compared to WT mice using arterial spin labeling (ASL). We used a similar method to that reported previously (Kober et al., 2008; Duncombe et al., 2017b) which overestimates perfusion values and thus determined the % changes in CBF in BCAS compared to baseline (Figure 2) (note: absolute values are shown in Supplementary Figure 1). The % reductions in regional CBF in the dorsolateral cortex and hippocampal CA1-DG region in BCAS compared to sham mice are shown (Figure 2A). In the dorsolateral cortex (DL CTX), there was a significant main effect of surgery [*F*(1, 26) = 14.816, *p* < 0.001] but no effect in Tg-SwDI mice (*p* > 0.05) on resting CBF (Figure 2B). *Post hoc* analysis indicated that CBF was significantly reduced in BCAS mice in both wild-type (*p* = 0.013) and Tg-SwDI (*p* = 0.010) groups. Furthermore, in the hippocampal CA1-DG region, there was a main effect of surgery [*F*(1, 26) = 17.963, *p* < 0.001] but not genotype (*p* > 0.05) (Figure 2C). *Post hoc* analysis showed significantly reduced CBF in both wild-type (*p* = 0.022) and Tg-SwDI (*p* = 0.002) BCAS groups. Although there was a sustained and prominent reduction in CBF, there were no genotype differences indicating that perfusion was reduced to a similar extent in WT and Tg-SwDI mice.

### Bilateral Common Carotid Stenosis Causes a Decline in Spatial Learning Acquisition and Cognitive Flexibility in Wild-Type and Tg-SwDI Mice

We and others have previously reported that BCAS caused short-term spatial working memory impairments and spatial learning and memory deficits in WT mice (Shibata et al., 2007; Coltman et al., 2011; Holland et al., 2015; Matin et al., 2016; Kitamura et al., 2017; Patel et al., 2017). In this study spatial learning and memory abilities were assessed in BCAS wild-type mice but additionally it was determined whether BCAS would cause an exacerbated impairment in Tg-SwDI mice. The experimental paradigm is outlined in Figure 1A. The speed or velocity was measured at the outset to assess whether motor function was affected by the genotype or surgery across different groups. There was a significant effect of genotype between WT and Tg-SwDI mice [*F*(1, 30) = 8.239, *p* < 0.01] (Figure 1B). *Post hoc* tests showed significant difference between WT BCAS and Tg-SwDI BCAS mice at day 1 (*p* < 0.01), 3, 4, and 5 (*p* < 0.05), respectively. The Barnes maze paradigm was used to evaluate visuo-spatial learning whereby mice were trained to locate an escape hole using spatial cues over 6 days with 2 sessions per day and the escape latency measured. There was a significant effect of BCAS [*F*(1, 30) = 9.60, *p* < 0.01] but not genotype (*p* > 0.05) on escape latency (Figure 1C). *Post hoc* tests showed a significant effect of BCAS in both WT and Tg-SwDI mice when compared to their sham counterparts at day 5, 6 (*p* < 0.05) and day 2 (*p* < 0.05), respectively. We next analyzed the distance traveled in the tests (pathlength) as an additional measure to evaluate spatial learning. There was a significant effect of BCAS [*F*(1, 30) = 5.826, *p* = 0.022] but no effect of genotype (*p* > 0.05) on the pathlength across groups (Figure 1D). *Post hoc* tests showed a significant effect of BCAS in WT mice when compared to their sham counterparts at day 1, 2, 5 (*p* < 0.05) and 6 (*p* < 0.01). To investigate the effect of BCAS on long-term memory, a probe test was taken after 72 h of the final acquisition training to examine whether experimental animals remember the previous target location after removing the escape chamber. Data was quantified as the percentage of time each mouse spent in the target quadrant where the allocated chamber was previously located. WT sham (*p* = 0.004), Tg-SwDI sham (*p* = 0.021) and Tg-SwDI BCAS (*p* = 0.025) all spent a significantly higher percentage of time than chance (25%) in the target quadrant but wild-type BCAS mice, did not perform above chance level (*p* = 0.84) (Figure 1E). There was no significant effect of BCAS or genotype on the percentage of time spent in the correct quadrant across groups (*p* > 0.05, respectively). To enhance the detection of spatial learning ability, reversal training and probe trials were then undertaken to evaluate the ability of experimental animals to learn a new location and to test cognitive flexibility. The escape hole location was switched 180° to the opposite side of maze. In the reversal tests, spatial learning was assessed by comparing escape latency and pathlength over 3 days with 2 sessions per day training across all groups. There was a significant effect of BCAS surgery [*F*(1, 30) = 4.70, *p* = 0.038] but not genotype by comparing escape latency (*p* > 0.05) (Figure 1F). *Post hoc* tests showed a significant effect of BCAS in WT mice when compared to their sham counterparts at day 2 (*p* < 0.05). By comparing the pathlength in the test, there was a significant effect of BCAS surgery [*F*(1, 30) = 5.84, *p* = 0.022] but not genotype (*p* > 0.05) (Figure 1G). *Post hoc* tests showed a significant effect of BCAS in WT mice when compared to their sham counterparts at day 2 (*p* < 0.05). The reversal probe was performed following 72 h after the final reversal training trial. Only WT sham mice (*p* < 0.05) spent a significantly higher percentage of time than chance in the target quadrant whereas all other groups did not perform above chance (WT BCAS, Tg-SwDI sham, Tg-SwDI BCAS *p* > 0.05, respectively) (Figure 1H). There was no significant effect of surgery (*p* > 0.05) or genotype (*p* > 0.05) on percentage time spent in the correct quadrant. Collectively the data demonstrate that BCAS impairs learning acquisition and cognitive flexibility.

### Cerebrospinal Fluid Glymphatic Influx in the Brain Cortex

**FIGURE 3:**
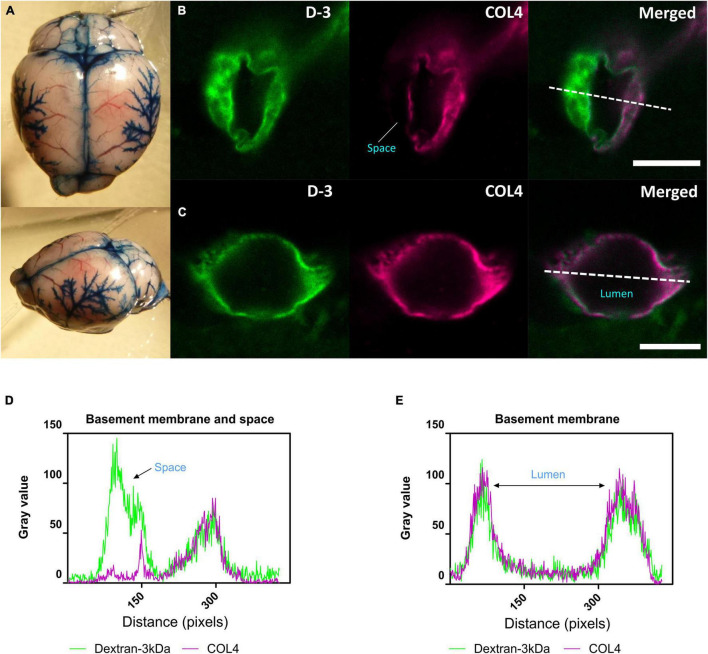
CSF tracer influx in the perivascular space. **(A)** Evans blue dye was injected into cisterna magna of a normal mouse. At the surface of the brain, dyes were found distributed along blood vessels, the middle cerebral artery (MCA) and its branches, along the superior sagittal sinus, inferior cerebral vein, and transverse sinus. **(B) (Arteriole, in cortical area)** and **(D)** Co-labeling of sections with the vascular basement membrane marker COL4 revealed the localization of CSF fluorescent tracer to the adjacent space. **(C) (Capillary, in subcortical area)** and **(E)** Tracer colocalized with the basement membrane. Representative images showing spatial location of tracer soluble lysine fixable dextran 3 kDa (D-3) (green). Scale bar: top (space) = 10 μm, bottom (lumen) = 5 μm.

We next determined whether glymphatic function would be impaired post-stenosis at a time when both cerebral perfusion and cognitive abilities are impaired. To address this, we first examined the distribution of Evans blue following cisterna magna injection as a method to visualize glymphatic entry/influx and found that the dye distributed along the surface brain vessels (e.g., middle cerebral artery), along the superior sagittal sinus, inferior cerebral vein, and transverse sinus (Figure 3A). Following this CSF influx was then investigated by injection of fluorescently labeled CSF tracer (Dextran 3 kDa, D-3) into the cisterna magna and tracer distribution evaluated by imaging *ex vivo* fixed brain slices labeled with a marker of the basement membrane (COL4). CSF tracer influx was observed colocalized with the basement membrane and in the adjacent space (Figure 3B), colocalized with basement membrane (Figure 3C). Intensity profile graphs show strong colocalization between CSF tracer (D-3) and vascular basement membrane (COL4) (Figures 3D,E) with partial tracer occupancy in the perivascular compartment (Figure 3D).

### Regional Cerebrospinal Fluid Tracer Influx Is Altered Post-bilateral Common Carotid Stenosis in Wild-Type and Tg-SwDI Mice

**FIGURE 4:**
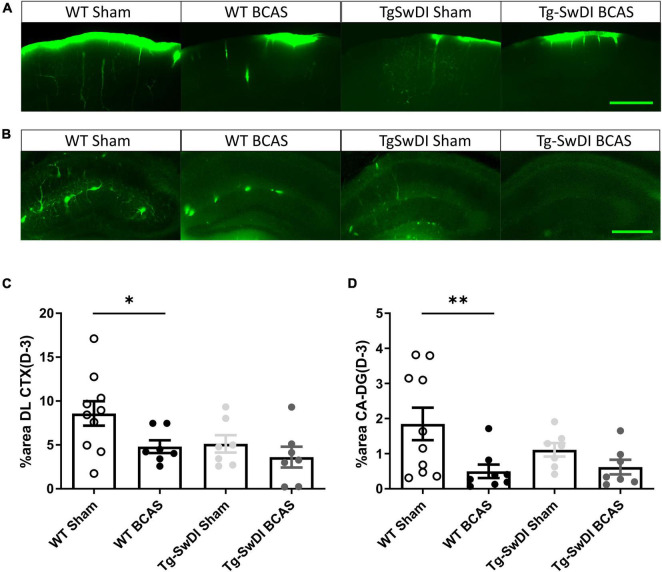
Regional CSF tracer influx is altered in BCAS and Tg-SwDI mice. Representative images of fluorescent tracer influx (D-3) (green) in the **(A)** DL CTX and **(B)** hippocampus (CA1-DG molecular layer) of WT and Tg-SwDI mice sham and post-BCAS. **(C,D)** Quantification of D-3 tracer distribution in the DL CTX and CA1-DG molecular layer. * and ^**^ indicate *p* < 0.05 and 0.01, respectively. Data are shown as individual data points, mean ± SEM, *n* = 6–10 per group. Scale bar = 500 μm.

The distribution of CSF tracer influx was then measured post-BCAS in both wild-type and Tg-SwDI mice. It was noted that the tracer distribution was quite heterogeneous between the different cohorts particularly in different brain regions, notably the dorsolateral cortex (DL CTX) and hippocampus (CA1-DG molecular layer). CSF tracer influx in the region of dorsolateral cortex was distributed along the middle cerebral artery (MCA) and its branches (Figure 3A) but this was less prominent post-BCAS compared to sham (Figure 4A). Quantification of tracer indicated a significant reduction in the dorsolateral cortex post-BCAS [*F*(1, 27) = 4.81, *p* = 0.037] and a trend toward an effect of genotype albeit this did not reach statistical significance (*p* = 0.064). *Post hoc* analysis showed a significant reduction in WT BCAS compared to sham animals (*p* < 0.05) (Figure 4C). It was also noted that CSF tracer was prominently distributed along the vascular network within the hippocampus but was markedly restricted post-BCAS in the hippocampal subregion: CA1-DG molecular layer (Figure 4B). It was determined that there was a reduction in tracer post-BCAS with a significant main effect of surgery [*F*(1, 28) = 7.5, *p* = 0.011], but no effect of genotype (*p* > 0.05). *Post hoc* tests showed a significant reduction between WT sham and BCAS mice (*p* = 0.005) (Figure 4D). Collectively, the results demonstrate that carotid stenosis has a major impact on cortical and hippocampal glymphatic function.

### Bilateral Common Carotid Stenosis Exacerbates Vascular Amyloid Accumulation

**FIGURE 5:**
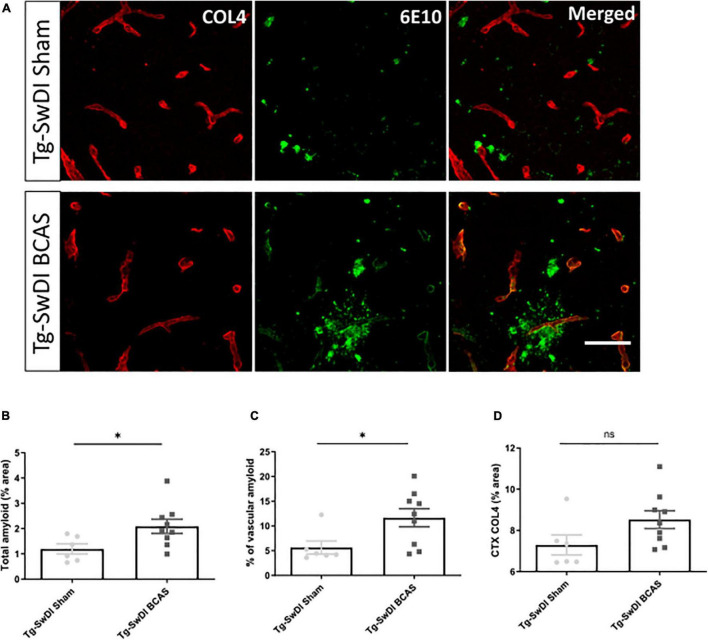
BCAS exacerbates amyloid deposition in Tg-SwDI mice. **(A)** Representative images of amyloid (green) and COL4 as a marker of vascular basement membranes (red) in the superficial brain cortex in Tg-SwDI sham and BCAS mice. **(B)** Total amyloid and **(C)** vascular amyloid were increased post-BCAS. **(D)** No significant changes of basement membrane were found. * indicates *p* < 0.05. Data are shown as individual data points, mean ± SEM, *n* = 6–9 per group. Scale bar = 50 μm.

The vascular basement membranes have been proposed as pathways for the movement of fluid in the brain and involved in the build-up of amyloid causing CAA (Morris et al., 2016). To investigate the potential changes of amyloid burden post-BCAS, we evaluated Aβ (6E10) load in the cortex and co-labeled with COL4 (a marker of basement membrane of blood vessels) to enable the assessment of microvascular amyloid in our Tg-SwDI mouse model with vascular amyloidosis (Figure 5A). A significant increase in the total amount of amyloid (*p* < 0.05) and vascular amyloid was determined post-stenosis (*p* < 0.05) in the cortex (∼250 μm from the pial surface) (Figures 5B,C, respectively). Since basement membranes have been shown as pathways for the clearance of Aβ we further determined COL4 levels but did not find significant changes post-BCAS (*p* > 0.05) (Figure 5D).

### Increased Astrogliosis Following Bilateral Common Carotid Stenosis in Cortex

**FIGURE 6:**
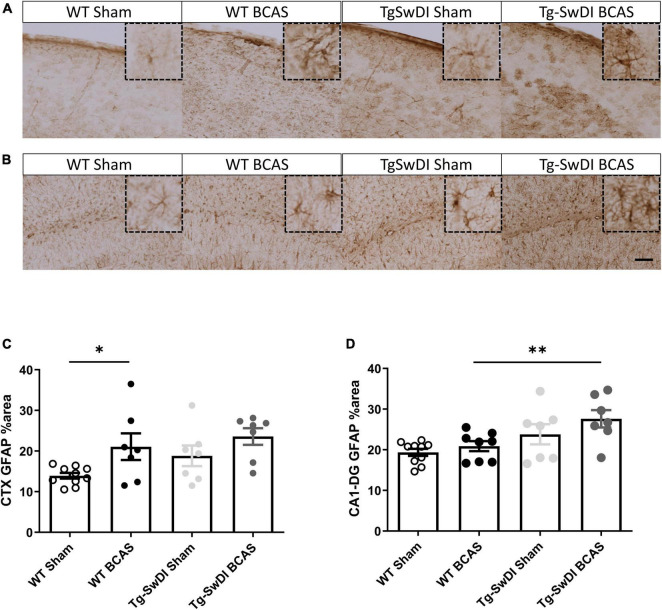
Increased astrogliosis post-BCAS in cortex. Representative images of GFAP immunostaining to assess the degree of astrogliosis in the superficial brain cortex of WT and Tg-SwDI, sham and BCAS mice. **(A,C)** In the superficial cortex, BCAS caused increased astrogliosis but was unaffected in Tg-SwDI mice. **(B,D)** In the hippocampus, there was increased astrogliosis in Tg-SwDI mice but not post-BCAS. * and ^**^ indicate *p* < 0.05 and 0.01, respectively. Data are shown as individual data points, mean ± SEM, *n* = 6–10 per group. Scale bar = 100 μm.

To discern the mechanisms by which BCAS may impact on glymphatic function we next studied the extent of astrogliosis. Astrocytes and their end-feet have been shown to alter glymphatic function (Iliff et al., 2012). GFAP immunostaining was undertaken to investigate the extent of reactive gliosis post-BCAS and in Tg-SwDI mice. BCAS surgery had a significant effect [*F*(1, 27) = 0.309, *p* = 0.01] but there was no effect of genotype (*p* > 0.05) on the extent of astrogliosis in the dorsolateral cortex. *Post hoc* tests showed a significant increase of astrogliosis between WT sham and BCAS mice (*p* = 0.021) (Figures 6A,C). We further analyzed the hippocampal CA1-DG molecular layer. There was a significant effect of genotype [*F*(1, 28) = 0.457, *p* = 0.002] but no effect of BCAS (*p* > 0.05) on astrogliosis. *Post hoc* tests showed a significant increase of astrogliosis in Tg-SwDI BCAS mice compared to WT BCAS group (*p* = 0.009) and a trend of increased astrogliosis between the WT sham and Tg-SwDI mice (*p* = 0.061) (Figures 6B,D). Thus, alterations in astrogliosis did not always parallel the impairment in glymphatic function observed post-BCAS.

### Cerebral Arterial Pulsation Is Impaired in Tg-SwDI and Post-bilateral Common Carotid Stenosis in Subset of Vessels

**FIGURE 7:**
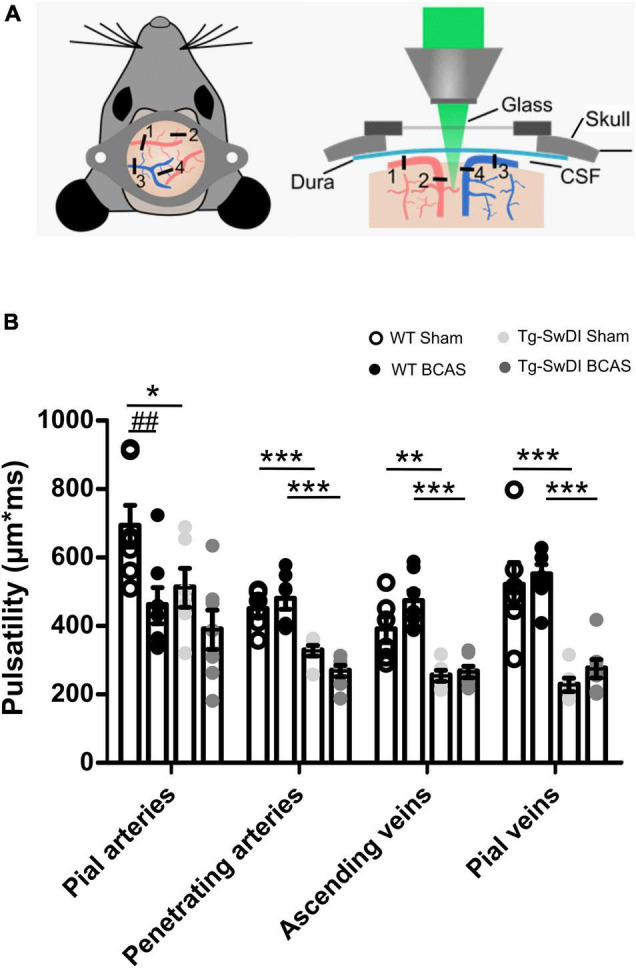
Vascular pulsatility is reduced in Tg-SwDI animals **(A)** Two-photon microscopy was used to assess vessel pulsation in sham and post-BCAS WT and Tg-SwDI mice. Four categories of blood vessels were investigated: 1. Pial arteries; 2. Penetrating arteries; 3. Ascending veins; 4. Pial veins. **(B)** There was a significant effect of genotype on vascular pulsatility in all types of vessels measured, and significant effect of surgery on pial arteries. *Post hoc* test revealed a significant decrease in vascular pulsation between wild type and Tg-SwDI sham animals in pial veins (*p* = 0.000), pial arteries (*p* = 0.041), penetrating arteries (*p* = 0.001) as well as ascending veins (*p* = 0.002) Vascular pulsation was also reduced in Tg-SwDI when compared to wild type BCAS mice in pial veins (*p* = 0.000), penetrating arteries (*p* = 0.000), ascending veins (*p* = 0.000) but not in pial arteries (*p* > 0.05). Finally, vascular pulsation was decreased in wild type BCAS animals when compared to shams (*p* = 0.008). Data are presented as mean ± SEM, *n* = 6–7 per group. ^#^ indicates significant difference between sham and BCAS; * indicates significant difference between WT and Tg-SwDI. *p < 0.05, **, ^##^*p* < 0.01; ***p < 0.001.

Cerebral arterial pulsation is thought to drive the glymphatic influx into and through the brain and is essential for the clearance of Aβ (Iliff et al., 2013b; Kress et al., 2014). Most recent evidence using two-photon imaging has supported the role of arterial pulsations in CSF movement in the perivascular spaces and basement membranes (Mestre et al., 2018). To investigate whether vascular pulsation is affected in Tg-SwDI animals and post-BCAS, we used *in vivo* two-photon microscopy which provides high temporal resolution of individual blood vessels. We measured multiple levels of the cerebral vascular network including pial veins and arteries, penetrating arteries and ascending veins (Figure 7A). There was a significant effect of genotype on vascular pulsatility of vessel diameter in all types of vessels measured (Figure 7B): pial veins [*F*(1, 22) = 53.841, *p* = 0.000], pial arteries [*F*(1, 23) = 4.744, *p* = 0.04], penetrating arteries [*F*(1, 22) = 46.398, *p* = 0.000] and ascending veins [*F*(1, 22) = 40.658, *p* = 0.000]. *Post hoc* tests revealed a significant decrease in vascular pulsation between wild type and Tg-SwDI sham animals in pial veins (*p* = 0.000), pial arteries (*p* = 0.041), penetrating arteries (*p* = 0.001) as well as ascending veins (*p* = 0.002). Vascular pulsation was also reduced in Tg-SwDI when compared to wild type BCAS mice in pial veins (*p* = 0.000), penetrating arteries (*p* = 0.000), ascending veins (*p* = 0.000), but not in pial arteries (*p* > 0.05). Interestingly, we found a significant effect of surgery in pial arteries only [*F*(1, 23) = 9.536, *p* = 0.005], but not in any other type of vessel. *Post hoc* analysis showed a significant decrease in vascular pulsation in wild type BCAS animals when compared to shams (*p* = 0.008), but no effect in Tg-SwDI animals (Figure 7B). In addition, heart rate was measured during imaging in all animals and was found to be similar across all groups (Supplementary Figure 2). Thus the decreased pulsatility is likely not driven by changes in frequency but rather by decreases in amplitude.

## Discussion

**FIGURE 8:**
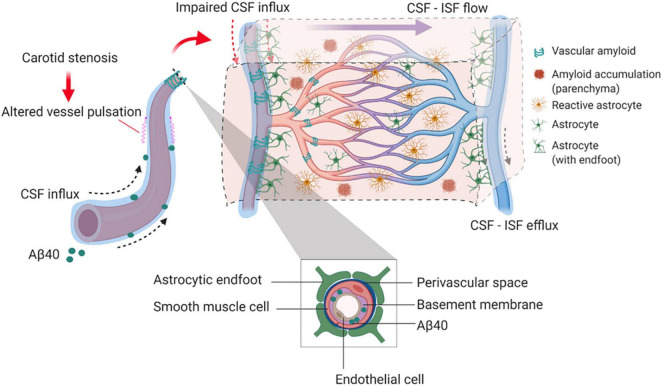
Proposed model by which BCAS and amyloid may impact on glymphatic function and predispose to VCI. In the long-term response to BCAS, reduced arterial pulsatility may impede CSF influx along the periarterial space and contribute in part to amyloid accumulation within the parenchyma and vasculature. Independently amyloid (Aβ40) has a profound impact on arterial pulsatility and impedes CSF influx. Downstream these events may influence glial cell responses including reactive astrogliosis and inflammation that can contribute to VCI.

Our findings provide experimental evidence that long-term BCAS, whilst reducing cerebral perfusion, may also affect glymphatic function (summarized in Figure 8). This new data adds credence to a growing body of human studies that have challenged the view that reduced blood flow post-stenosis is the major contributor to VCI. Instead, alternative or additional mechanisms should be considered (Aribisala et al., 2014a; Wardlaw et al., 2017; Alhusaini et al., 2018; Shi et al., 2018a).

A substantial number of studies have shown that BCAS, using microcoils applied to both common carotid arteries in mice, leads to cognitive impairment (Shibata et al., 2007; Matin et al., 2016; Patel et al., 2017). Consistent with these studies, we show that at 3 months after BCAS, impaired spatial learning acquisition and cognitive flexibility are evident. The mechanistic link between BCAS and VCI has largely been attributed to the post-BCAS cerebral perfusion deficits initiating hypoxia–induced white matter pathology and degenerative changes to the glial-vascular unit (Holland et al., 2015).

Our novel data suggest that BCAS can also lead to impaired CSF influx along the glymphatic pathway. In the first instance, we injected fluorescent tracer into the cisterna magna and were able to observe the tracers surrounding cerebral arteries e.g., middle cerebral artery (MCA) within the perivascular compartment (Figure 3) consistent with previous observations showing that CSF influx moves along the periarterial components into deeper brain regions (Iliff et al., 2012). It has been shown by *in vivo* two-photon imaging that intracisternal CSF tracer travels along the perivascular component surrounding pial surface (Iliff et al., 2012; Xie et al., 2013). Using confocal microscopy, we were able to measure the regional distribution of tracer post-BCAS. Following 3 months of carotid stenosis impaired glymphatic function was determined in cortical and deep hippocampal regions suggesting that prolonged disruption to the vascular system may lead to enduring suppression of CSF influx to the brain.

CSF influx along the glymphatic drainage pathway has also shown to be impaired in other models relevant to cerebral vascular disease. In a rodent model of multiple infarcts, caused by intra-arterial injection of cholesterol crystals via the internal carotid artery, a transient suppression of CSF influx was determined (Wang et al., 2017). However, in this study glymphatic function was restored within 2 weeks. In other models a sustained or progressive impairment of glymphatic function has been shown such as with aging (Wang et al., 2017), hypertension (Mestre et al., 2018) and in models relevant to AD (Peng et al., 2016). Interestingly, in another study of APP/PS1 mice, a model relevant to AD, glymphatic function is severely compromised before amyloid accumulation (Peng et al., 2016) and it is likely that there are different pathophysiological mechanisms that can contribute to VCI.

To examine potential mechanisms underlying the impaired CSF influx, we assessed the extent of astrogliosis as astrocytes have an important function in CSF influx and clearance (Iliff et al., 2012), and deletion of the astrocytic end feet reduces the CSF influx into the parenchyma following ischemia (Mestre et al., 2020). In this study there was a tendency for astrogliosis post-BCAS. In the cortex there was pronounced astrogliosis most notable in WT mice post-BCAS and in the hippocampus astrogliosis was increased in Tg-SwDI post-BCAS mice. We further explored additional mechanisms that may account for the impaired CSF influx post-BCAS. Cerebrovascular pulsatility is a key driving force facilitating CSF flow into and through brain parenchyma (Iliff et al., 2013b; Mestre et al., 2018) and vasoconstriction was shown previously to play an important role in CSF flow following ischemia (Mestre et al., 2020). Using intravital imaging we found that arterial pulsation in pial vessels was affected in transgenic animals and was further exacerbated by BCAS surgery. Our results are in accordance with previous data showing that 30 min of unilateral ligation of internal carotid artery leads to significantly reduced pulsatility in the penetrating arteries with impaired glymphatic influx (Iliff et al., 2013b). Our data shows that venous pulsation, on the other hand, as well as pulsation of penetrating and ascending vessels was impaired in transgenic animals, but not affected by BCAS surgery. Together, these data suggest that BCAS has a differential effect on vascular pulsation within the vascular bed with arterial pulsation predominantly exacerbated by BCAS. Since cerebral pulsation may govern CSF-ISF exchange in murine brain leading to accumulation of solutes/proteins in the brain, this impairment may partly explain the accumulation of Aβ that we determined in Tg-SwDI animals post-BCAS. Altered carotid function has been associated with impaired cognitive function, greater Aβ deposition and several features of vascular disease in both human and animal studies (Huang et al., 2012; Poels et al., 2012; Ding et al., 2015; Holland et al., 2015; Hughes et al., 2018). It has been also shown that the impaired glymphatic function can result in increased Aβ burden in the brain (Kress et al., 2014; Peng et al., 2016; Shokri-Kojori et al., 2018). In the current study arterial pulsation was impaired by BCAS and independently by Aβ in Tg-SwDI in the absence of BCAS. It has been suggested that arterial vessel stiffening and reduced pulsatility may cause decreased CSF fluid influx (Benveniste and Nedergaard, 2021) and thus BCAS/Aβ accumulation may impact on vascular hemodynamics resulting in altered glymphatic function. In keeping with this, in studies of human SVD, pulsatility index as a measure of vascular stiffness is increased (Shi et al., 2018b). However of note in this study is, that although BCAS led to an increase in Aβ accumulation, this was not associated with a more prominent impairment in glymphatic function. One explanation may be that glymphatic function is already markedly reduced in Tg-SwDI mice. Alternatively there may be other mechanisms that contribute to amyloid accumulation post-BCAS (e.g., oxidative stress), which we have previously shown (Salvadores et al., 2017). A prospective population-based study has also shown alterations of pulsation in the carotid artery may contribute to the pathophysiology of cerebral microbleeds in deep brain region secondary to hypertension (Ding et al., 2015). Interestingly we have also shown that sustained carotid stenosis leads to the development of vascular lesions (both microbleeds and microinfarcts) in deep subcortical structures several months post-stenosis (Holland et al., 2015).

The present study was restricted to evaluation of CSF influx at 3 months post-BCAS and future longitudinal approaches to evaluate potential progression of changes could be interrogated using contrast-enhanced MRI (Iliff et al., 2013a). One of the limitations of the present study was the inability to intervene and modify glymphatic function. Thus in future it will be important to discern whether glymphatic failure can be reversed and whether this may impact on the onset and progression of VCI.

## Data Availability Statement

The original contributions presented in the study are included in the article/Supplementary Material, further inquiries can be directed to the corresponding author: (Karen.Horsburgh@ed.ac.uk).

## Ethics Statement

The animal study was reviewed and approved by the UK Home Office Animals (Scientific Procedures) Act 1986 and additional local ethical and veterinary approval (Biomedical Research Resources, University of Edinburgh).

## Author Contributions

ML carried out, designed, analyzed most of the experiments, and wrote the manuscript. AK carried out and designed the CSF tracer experiment. JB carried out multiphoton experiments, data analysis, and some behavioral work. JK carried out multiphoton experiment and CSF tracer experiment analysis. JD carried out MR imaging and behavioral and IHC experiment. RL, MJ, and IM overseen the MR/ASL imaging and analysis. BP assisted with the behavioral study design. UW assisted with the multiphoton work. RC assisted the CSF tracer experiment design, data interpretation, and editing manuscript. RK assisted the data interpretation, and editing manuscript. JI assisted the study design, data interpretation, and editing manuscript. KH conducted the surgeries, supervised the project and assisted in study design, data interpretation, and writing of the manuscript. All authors assisted with editing of the manuscript.

## Conflict of Interest

The authors declare that the research was conducted in the absence of any commercial or financial relationships that could be construed as a potential conflict of interest.

## Publisher’s Note

All claims expressed in this article are solely those of the authors and do not necessarily represent those of their affiliated organizations, or those of the publisher, the editors and the reviewers. Any product that may be evaluated in this article, or claim that may be made by its manufacturer, is not guaranteed or endorsed by the publisher.

## Abbreviations

(A β): Amyloid- β
ASL: Arterial spin labeling
ACSF: Artificial cerebrospinal fluid
BCAS: Bilateral common carotid stenosis
CAA: Cerebral amyloid angiopathy
CBF: Cerebral blood flow
CVD: Cerebral vascular disease
DG: Dentate gyrus
DL CTX: Dorsolateral cortex
MCA: Middle cerebral artery
PVS: Perivascular space
PBS: Phosphate-buffered saline
ROI: Region of interest
SAH: Subarachnoid hemorrhage
Tg-SwDI: Transgenic mouse containing the Swedish, Dutch and Iowa mutations
VCI: Vascular cognitive impairment
WT: Wild-type.
