# Supplementary material for: Impaired Glymphatic Function and Pulsation Alterations in a Mouse Model of Vascular Cognitive Impairment
Source: Front Aging Neurosci. 2022 Jan 13;13:788519. doi: 10.3389/fnagi.2021.788519 (PMC8793139; doi:10.3389/fnagi.2021.788519)
Supplement: Supplementary file 1 [file Data_Sheet_1.PDF]

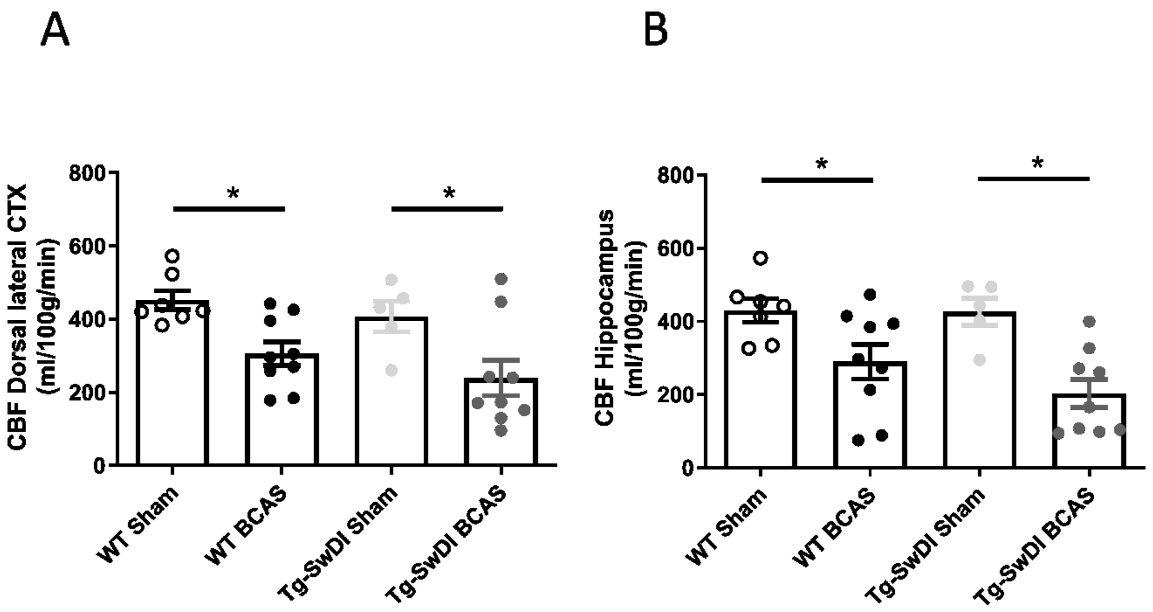

Supplementary Figure 1

Absolute CBF values were measured using MRI arterial spin labelling (ASL) and regional alterations in CBF were assessed in the dosolateral cortex (DL CTX) and hippocampus. **A** There was a significant effect of BCAS in the DL CTX (F (1, 26) =14.816, p<0.001) but no effect of genotype (p>0.05), post-hoc analysis indicated that CBF was significantly reduced in BCAS mice in both wild-type (p=0.013) and Tg-SwDI (p=0.010) groups. **B** Similarly in the hippocampus there was a significant effect of surgery (F (1, 26) =17.963, p<0.001) but no effect of genotype (p>0.05). Post-hoc analysis indicated there was a significant reduction of CBF in the brain DL CTX and hippocampus post-BCAS in both WT and Tg-SwDI mice. * indicates p<0.05. Data are presented as mean ± SEM, n=6-10 per group.

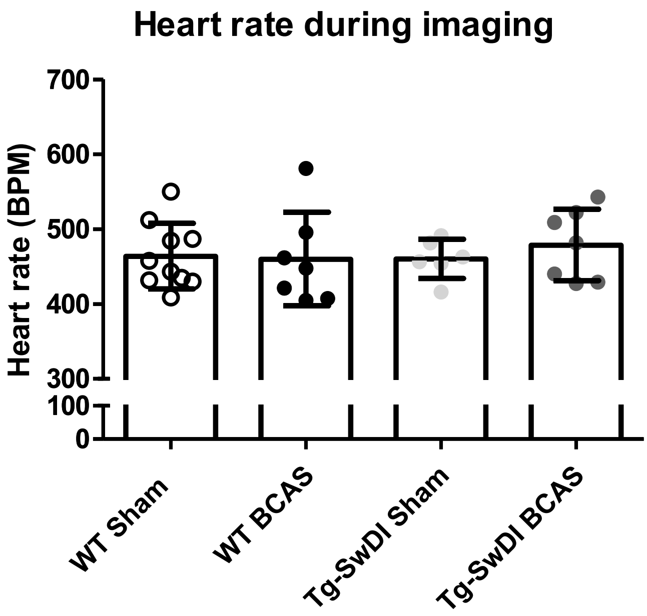

Supplementary Figure 2

Heart rate values (BPM) of all experimental groups (WT sham, WT BCAS, Tg-SwDI Sham and Tg-SwDI BCAS) were measured throughout imaging at 10 minute intervals using MouseSTAT foot pad heart monitor. No significant differences were found for either genotype ($F_{(1,23)} = 0.171$) or surgery ($F_{(1,23)} = 0.227$).

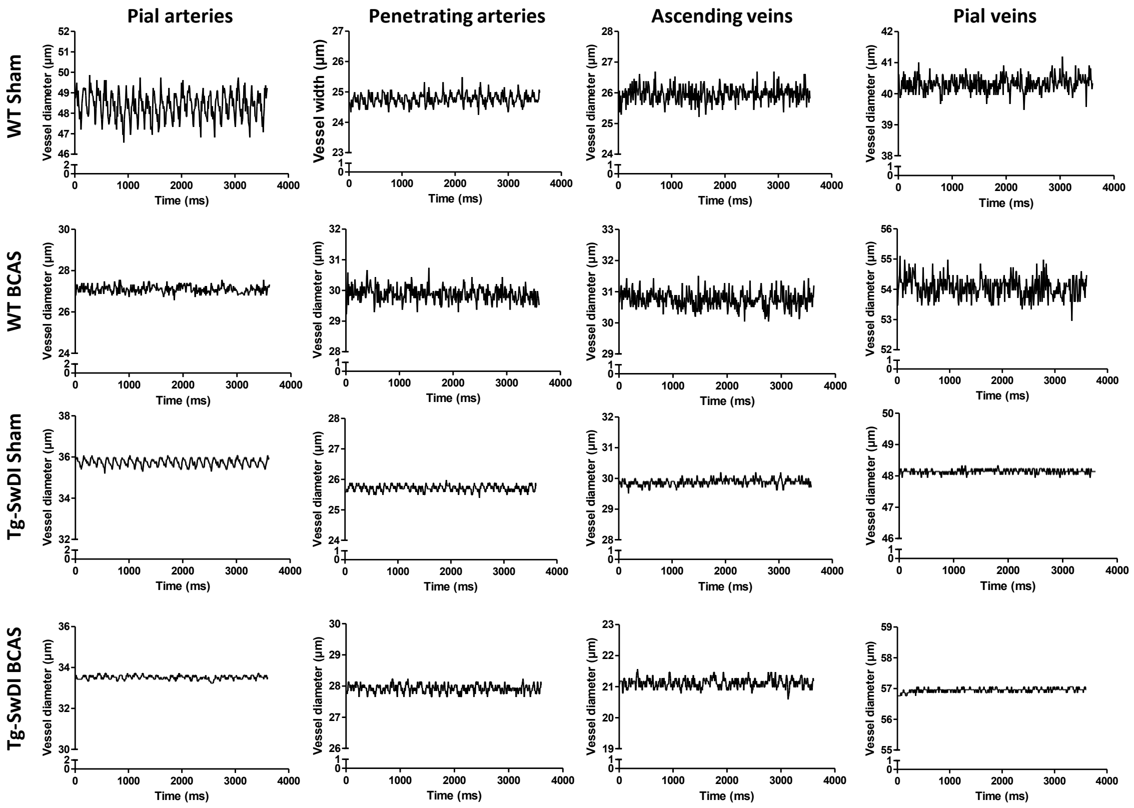

Supplementary Figure 3

Representative images of raw vessel diameter time profiles to investigate vascular pulsation and pulsation frequency. Data shown represents profiles from all four types of vessels imaged (pial arteries, penetrating arteries, ascending veins and pial veins) and of all four groups (WT Sham, WT BCAS, Tg-SwDI Sham and Tg-SwDI BCAS). Total pulsation was calculated as area under curve of the absolute value of diameter integrated over running average across the sampling time. Pulsation frequency was calculated as the total of positive and negative peaks from the same graphs.
